# Assessing the Impact of Pre-Existing Mental Health and Neurocognitive Disorders on the Mortality and Severity of COVID-19 in Those Aged Over 18 Years: A Systematic Review and Meta-Analysis

**DOI:** 10.1192/bjo.2022.253

**Published:** 2022-06-20

**Authors:** Catrin Thomas, Laura Williams, Asha Dhandapani, Sarmishtha Bhattacharyya

**Affiliations:** 1 Betsi Cadwaladr University Health Board, Ruthin, United Kingdom.; 2 Betsi Cadwaladr University Health Board, Rhyl, United Kingdom; 3 Betsi Cadwaladr University Health Board, Wrexham, United Kingdom.; 4 University of Chester, Chester, United Kingdom

## Abstract

**Aims:**

Since the coronavirus disease 2019 (COVID-19) pandemic began, evidence suggests that people with underlying mental health disorders have worse outcomes from COVID-19 infection. Our aim was to assess the impact of COVID-19 infection on people with pre-existing mental health or neurocognitive disorder including COVID-19 related mortality and severity.

**Methods:**

We conducted systematic searches of PubMed, EMBASE, and Cochrane library for articles published between 1 December 2019 and 15 March 2021. The language was restricted to English. We included all case control, cohort and cross sectional studies that reported raw data on COVID-19 associated mortality and severity in participants aged 18 years or older with a pre-existing mental health or neurocognitive disorder compared to those without. Three independent reviewers extracted data according to the Preferred Reporting Items for Systematic Reviews and Meta-Analyses (PRISMA) and the Meta-analysis of Observational Studies in Epidemiology (MOOSE) guidelines. Methodological quality and risk of bias were assessed using the 9-star Newcastle-Ottawa Scale. We calculated the odds ratio as the summary measure along with the corresponding 95% confidence intervals. The random effects model was used to calculate the overall pooled risk estimates. COVID-19 related mortality was the primary outcome measure. The secondary outcome measure was COVID-19 related severity, defined as intensive care unit admission or use of mechanical ventilation.

**Results:**

Fifteen studies were included in the meta-analysis comprising of 8,021,164 participants. There was a statistically significant increased risk of mortality for participants with a pre-existing mental health or neurocognitive disorder compared to those without (OR = 2.18, 95% CI = 1.63–2.90, P < .00001). Increased mortality risk was found on subgroup analysis for participants with pre-existing schizophrenia (OR = 2.55, 95% CI = 1.38–4.71, P = .003) and dementia (OR = 3.83, 95% CI = 2.42–6.06, P < .00001). There was no statistically significant difference in the severity of illness when comparing the two groups. There was a statistically significant increase in the number of participants with comorbid diabetes and chronic lung disease in those with a pre-existing mental health or neurocognitive disorder compared to those without.

**Conclusion:**

The results show that people over 18 years with a pre-existing mental health or neurocognitive disorder have an increased risk of mortality from COVID-19 and are more likely to have comorbid diabetes and chronic lung disease. These results highlight the need for better physical health monitoring and management for this group of people and better integration of mental and physical health services, as well as adding to the evidence that they should be prioritised in the ongoing COVID-19 vaccination schedules worldwide.

